# Functional network connectivity of the control and epileptic hippocampus

**DOI:** 10.1186/1471-2202-12-S1-K1

**Published:** 2011-07-18

**Authors:** Ivan Soltesz

**Affiliations:** 1 Department of Anatomy & Neurobiology, University of California, Irvine, USA

## 

With the rapid rise in our knowledge about the structural and functional properties of hippocampal microcircuits, it has become possible to closely integrate experimental findings with large-scale, anatomically and biophysically realistic computational simulations of control and epileptic neuronal networks with unprecedented precision and predictive power. We are developing full-scale realistic network models of the control and injured temporal lobe in order to investigate fundamental questions related to normal hippocampal microcircuit function and the mechanistic bases of epilepsy. I review the conceptual framework and biological basis of model development and show specific applications, including new computational and experimental results concerning the phase-related firing of various interneuronal subtypes during learning and memory-related hippocampal network oscillations and the roles of aberrant hyper-connected hub-like neurons in seizures. The talk will highlight the unprecedented predictive and analytic power of increasingly user-friendly, freely shared, highly realistic, large-scale computational models in understanding normal circuit function and temporal lobe epilepsy.

